# Sodium chloride accumulation in glycophyte plants with cyanobacterial symbionts

**DOI:** 10.1093/aobpla/plx053

**Published:** 2017-10-11

**Authors:** Thomas George Allan Green, Leopoldo G Sancho, Ana Pintado, Dolores Saco, Soledad Martín, María Arróniz-Crespo, Miguel Angel Casermeiro, Maria Teresa de la Cruz Caravaca, Steven Cameron, Ricardo Rozzi

**Affiliations:** Departamento de Biología Vegetal II, Facultad de Farmacia, Universidad Complutense, 28040 Madrid, Spain; Biological Sciences, Waikato University, 3216 Hamilton, New Zealand; Departamento de Química y Tecnología de Alimentos, Universidad Politécnica de Madrid, 28040 Madrid, Spain; Departamento de Edafología, Facultad de Farmacia, Universidad Complutense, 28040 Madrid, Spain; Department of Chemistry, University of Waikato, 3216 Hamilton, New Zealand; Department of Philosophy and Religion Studies, University of North Texas, Denton, TX 76201, USA

**Keywords:** *Azolla*, chloride, cyanobacterium, *Gunnera*, salt, sodium, symbiosis

## Abstract

The majority of plant species are glycophytes and are not salt-tolerant and maintain low sodium levels within their tissues; if^.^ high tissue sodium concentrations do occur, it is in response to elevated environmental salt levels. Here we report an apparently novel and taxonomically diverse grouping of plants that continuously maintain high tissue sodium contents and share the rare feature of possessing symbiotic cyanobacteria. Leaves of *Gunnera magellanica* in Tierra del Fuego always had sodium contents (dry weight basis) of around 4.26 g kg^−1^, about 20 times greater than measured in other higher plants in the community (0.29 g kg^−1^). Potassium and chloride levels were also elevated. This was not a response to soil sodium and chloride levels as these were low at all sites. High sodium contents were also confirmed in *G. magellanica* from several other sites in Tierra del Fuego, in plants taken to, and cultivated in Madrid for 2 years at low soil salt conditions, and also in other free living or cultivated species of *Gunnera* from the UK and New Zealand. *Gunnera* species are the only angiosperms that possess cyanobacterial symbionts so we analysed other plants that have this rather rare symbiosis, all being glycophytes. Samples of *Azolla*, a floating aquatic fern, from Europe and New Zealand all had even higher sodium levels than *Gunnera*. Roots of the gymnosperm *Cycas revoluta* had lower sodium contents (2.52 ± 0.34 g kg^−1^) but still higher than the non-symbiotic glycophytes. The overaccumulation of salt even when it is at low levels in the environment appears to be linked to the possession of a cyanobacterial symbiosis although the actual functional basis is unclear.

## Teaser

We have found a novel group of plants that appear to obligately accumulate salt (sodium chloride) although the level in their environment is low. The group is taxonomically diverse including angiosperms (Gunnera spp.), gymnosperms (Cycas) and a fern (Azolla species) but all of them form a symbiosis with a cyanobacterium (blue-green alga) that carries out nitrogen fixation, a rare occurrence in plants. The functional basis for the high salt contents is not clear and, despite the high internal salt levels, the plants are unable to grow in high salt environments.

## Introduction

The vast majority of plants, 98–99 %, are glycophytes and are intolerant to salt (sodium chloride in this paper) and are unable to successfully grow in saline soils (Aslam *et al.* 2011). Glycophytes typically exclude sodium and maintain low sodium levels in their tissues at around 0.2 to 2.0 g kg^−1^ (Volkmar *et al.* 1998; Glenn *et al.* 1999; Rahdari and Hoseini 2011). Many species appear to preferentially take up potassium so that Na:K ratios are low (Flowers and Colmer 2008). A small number of glycophytes, when challenged with saline conditions, can develop some tolerance and have higher levels of sodium in their tissues (Munns and Tester 2008; Meychik *et al.* 2013) but in these cases the sodium content in their tissues is positively related to external levels. In contrast, halophytes are plants that survive and reproduce in environments where the external salt concentration is around 200 mM NaCl or more (Flowers and Colmer 2008) and constitute about 1 % of the world’s flora. Halophytes typically increase salt concentration in their cells to reduce water potential allowing pumping water from the soil. Halophytes employ a variety of methods to overcome the problems of excess of salinity. Many exclude or excrete the salt whilst others can have high levels of sodium in their tissues, for example 12 g kg^−1^ for sodium, and 35 g kg^−1^ for chloride in *Armeria maritima* (Kohl 1997). A few are obligate halophytes and require saline soils for successful growth.

Salinity reduces growth of sensitive plants via several quite distinct processes one of which is the accumulation of salt in the shoot (Munns 2002). Until now there appear to be no reported examples of plants that maintain high internal sodium (or salt because the predominant counter ion is chloride) in the absence of high external sodium levels.

**Figure 1.**
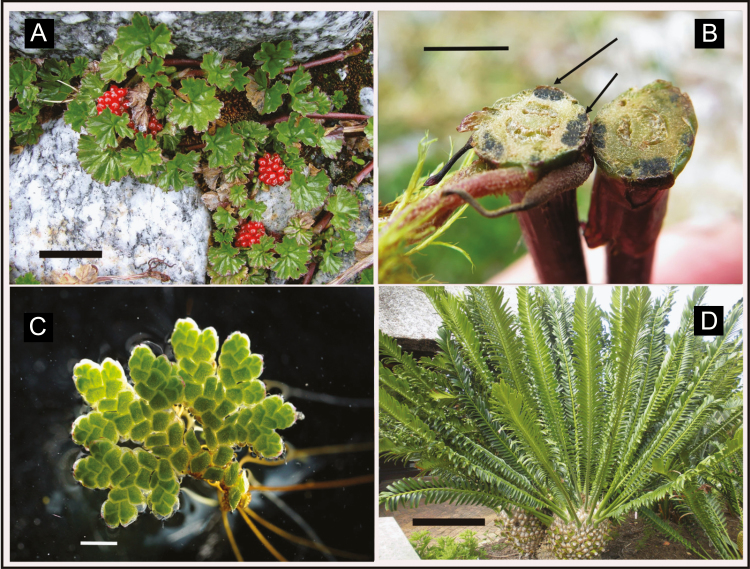
Pictures of plants analysed: A, *Gunnera magellanica* from Tierra del Fuego (bar is 5 cm); B, cross section of *G. magellanica* petiole bases showing cyanobacteria colonies (arrows point to colonies, bar is 1 cm); C, *Azolla filiculoides* (bar is 1 mm); D, *Cycas* species (bar is 30 cm).

When analysing the mineral contents of plants growing in front of receding Chilean glaciers, we discovered unusually high Na levels in the leaves of the locally common herbaceous angiosperm *Gunnera magellanica* (Fig. 1A). This occurred in the absence of elevated salt levels in the external medium and no other species growing in the same community with *G. magellanica* had elevated sodium levels in their leaves. The genus *Gunnera* is unusual in having symbiotic cyanobacteria in glands at the base of each petiole (Fig. 1B) which carry out nitrogen fixation (Silvester and Smith 1969; Osborne and Bergman 2009). We reasoned that if high accumulation of sodium is obligate in plants with symbiotic association with cyanobacteria, we should be able to detect this unusual trait in other plants with this symbiosis. We extended our analyses to plants that were taxonomically diverse with representatives from the angiosperms, gymnosperms and ferns and all sharing the unusual functional trait in having symbiotic cyanobacteria that provide fixed nitrogen (Rai *et al.* 2000; Santi *et al.* 2013).

## Methods

### Plant samples

All species tested are named with the sources of the samples used for analysis, in Table 1.

**Table 1.** Mean sodium, chloride and potassium contents of leaf tissues (g kg^−1^ ± 1 SE) and Na/K ratio.

Plants	Collection site	Mean concentration g kg^−1^ ± 1 SE			Na:K molar ratio ± 1 SE
		Na^+^	K^+^	Cl^−^	
Non-symbiotic					
*Gaultheria mucronata*	Pía Glacier chronosequence	0.30 ± 0.03	4.77 ± 0.47	14.50 ± 0.70	0.12 ± 0.01
*Nothofagus betuloides*	Pía Glacier chronosequence	0.23 ± 0.02	3.97 ± 0.50	15.23 ± 1.94	0.10 ± 0.02
*N. antarctica*	Pía Glacier chronosequence	0.37 ± 0.05	5.95 ± 0.27	20.29 ± 2.30	0.12 ± 0.02
Tierra del Fuego, Chile					
*Gunnera magellanica*	Pía Glacier chronosequence	4.41 ± 0.40	10.27 ± 0.70	26.99 ± 2.01	0.74 ± 0.05
	Parry Glacier chronosequence	3.99 ± 0.26	6.78 ± 1.69	32.08 ± 6.53	1.01 ± 0.05
	Puerto Williams – coast	8.95 ± 1.89	4.32 ± 0.80	25.52 ± 4.24	3.58 ± 0.34
	Puerto Williams – forest	8.90 ± 1.62	5.27 ± 0.65	16.32 ± 1.76	2.51 ± 0.39
	Puerto Williams – tundra	6.98 ± 1.42	7.17 ± 1.18	16.29 ± 3.37	1.69 ± 0.21
In greenhouse, Madrid					
Two years	Puerto Williams – coast	4.79 ± 0.18	8.46 ± 2.03	44.10 ± 5.86	1.01 ± 0.21
	Puerto Williams – forest	5.09 ± 0.13	3.29 ± 0.14	38.63 ± 5.11	2.65 ± 0.16
	Puerto Williams – tundra	3.98 ± 0.13	5.34 ± 0.04	40.18 ± 3.77	1.27 ± 0.07
*Gunnera* species from other locations					
*G. magellanica*	Cornwall, nursery	4.66 ± 0.18	11.15 ± 0.36	43.47 ± 8.58	0.71 ± 0.02
*G. magellanica*	Scotland, nursery	4.34 ± 0.27	9.43 ± 0.83	21.09 ± 6.48	0.78 ± 0.02
*Gunnera manicata*	Hamilton, New Zealand	3.56 ± 0.31	6.74 ± 0.37	25.78 ± 1.32	0.90 ± 0.07
*G. manicata*	Lamorna, Cornwall, England	7.61 ± 0.39	4.67 ± 0.24	51.63 ± 6.71	2.79 ± 0.25
*G. manicata*	Kew Gardens, London, England	6.23 ± 0.08	22.05 ± 0.76	21.09 ± 1.25	0.48 ± 0.01
*G. perpensa*	Kew Gardens, London, England	5.11 ± 0.26	6.19 ± 0.15	26.95 ± 4.04	1.40 ± 0.04
*G. hamiltonii*	Kew Gardens, London, England	4.58 ± 0.44	28.29 ± 0.64	51.03 ± 5.56	0.28 ± 0.02
*G. tinctoria*	Kew Gardens, London, England	3.68 ± 0.33	7.25 ± 0.50	19.66 ± 1.25	0.86 ± 0.02
*Azolla* samples					
*Azolla rubra*	Mangakahu, New Zealand	10.42 ± 0.30	18.51 ± 0.97	31.68 ± 3.89	0.96 ± 0.02
*A. rubra*	Invercargill, New Zealand	15.77 ± 4.19	25.74 ± 8.13	22.69 ± 7.10	0.89 ± 0.07
*A. rubra*	Hamilton, New Zealand	13.92 ± 1.41	17.80 ± 0.79	45.58 ± 2.78	1.33 ± 0.07
*A. filiculoides*	Cáceres, Spain	13.65 ± 0.46	17.10 ± 0.38	42.49 ± 2.55	1.36 ± 0.02
*Cycas revoluta*					
Coralloid root tissue.	Waikato University, New Zealand	2.52 ± 0.34	7.53 ± 0.30	14.95 ± 1.64	0.57 ± 0.06
Leaf tissue	Waikato University, New Zealand	0.53 ± 0.11	5.4 ± 0.68	0.44 ± 0.04	0.20 ± 0.04

#### Genus Gunnera.

*Gunnera magellanica* (Fig. 1A): The initial samples were collected along a chronosequence in front of the Pía Glacier on the south side of Mt. Darwin (54.7500°S, 69.4833°W), Cordillera Darwin in Tierra del Fuego, Chile. The surfaces produced by the retreat of this glacier have been accurately dated and a *Nothofagus* dominated forest has developed at the oldest site, only 34 years after exposure (Sancho *et al.* 2011). The developing vegetation is relatively simple with the most common plants being the shrub *Gaultheria mucronata*, the trees *Nothofagus antarctica*, *N. betuloides* and *G. magellanica* as ground cover. We collected five samples of mature leaves from each species when present at five sites of increasing surface age ranging from 3 to 34 years. Leaf samples from *Gunnera* spp. did not contain symbiotic cyanobacteria which occur at the base of the petiole.

Additional samples were collected along a second chronosequence in front of Parry Glacier (54.6833°S, 69.3833°W) on the north side of the Cordillera Darwin, and at three sites (near the coast, in forest [~300 m] and above tree line [~700 m]) near Puerto Williams, Navarino Island, Chile, which lies on the south shore of the marine seaway Beagle Channel (54.9333°S, 67.6167°W).

*Gunnera magellanica* plants from Puerto Williams were transplanted to greenhouses in Madrid and grown for 2 years watered with normal tap water: Fl < 100.00 µg L^−1^, CaCO_3_ < 45 mg L^−1^, Al < 0.1 mg L^−1^, free Cl < 0.1 mg L^−1^, combined Cl < 1.0 mg L^−1^, trihalomethanes < 30 mg L^−1^, pH 7–8, conductivity 100–200 µS cm^−1^ at 20 °C (www.madridsalud.es).

*Gunnera magellanica* plants were also purchased from commercial nurseries in Cornwall (England) and Scotland.

*Other Gunnera species*: leaf samples from *G. tinctoria*, *G. hamiltonii*, *G. perpensa* and *G. manicata* were obtained from the Royal Botanic Gardens, Kew, London (51.4746°N, 0.2955°W) and the latter species also from Lamorna Cove, Cornwall (50.0630°N, 5.5644°W; https://www.tripadvisor.com/LocationPhotoDirectLink-g1533350-d558744-i102032429-Lamorna_Cove-Lamorna_Penzance_ Cornwall_England.html) and Waikato University campus, Hamilton, New Zealand (37.7869°S, 175.3139°E).

#### Other plants with symbiotic cyanobacteria.

Coralloid root and leaf tissue of *Cycas revoluta* were obtained from the Biological Sciences plant collection, Waikato University (Fig. 1D); *Azolla rubra* samples were obtained from three sites in New Zealand, Hamilton (37.8015°S, 175.3300°E), Mangakahu Valley (38.7382°S, 175.4102°E) and Invercargill (46.4131°S, 168.3475°E). *Azolla filiculoides* (Fig. 1C) samples were collected in Cáceres, Spain (40.1306°N, 5.4639°W). *Azolla* samples were complete plats so that they contained both plant tissue and the symbiotic cyanobacteria. *Cycas* leaf tissue had no cyanobacteria present.

#### Non-symbiotic species.

Leaf samples of *Gaultheria mucronata*, *N. betuloides* and *N. antarctica* were collected from the Pía Glacier chronosequence at the same locations as the *G. magellanica* samples.

#### Sample treatment.

Leaf samples: leaf samples, at least 200 mg dry mass, from Tierra del Fuego, were taken from healthy leaves exposed to the sun, immediately oven dried (~60 °C) and stored dry until analysed.

### Leaf analysis

#### ICP/MS (inductively coupled plasma mass spectrometry).

Waikato University, Hamilton, New Zealand. Samples were digested in concentrated nitric acid on an aluminium heating block at 80 °C for an hour and then made up to a standard volume. In the ICP/MS measurements calibration blanks, flush blanks and calibration standards were compared against the samples and two certified reference materials were used (SLRS-5 and SLRS-4) of known concentrations to give a double check on the absolute concentration values produced. Every 24 samples the instrument was programmed to recalibrate and at the finish the data were drift corrected.

#### Ion chromatography.

Madrid: Samples were analysed in triplicate; all reagents used were of analytical grade (Merck, Darmstadt, Germany). Chloride was determined by ion chromatography (Chromatograph Metrohm 761 Compact IC) of deionized water extracts of ground dried leaf material (Liu 1998; Sadzawka *et al.* 2007). Ash from burning dry plant samples (muffle furnace at 500 °C) dissolved in HCl (50 %):HNO_3_ (50 %) (1:1; v/v) was analysed for mineral elements by atomic absorption spectrometry (AAS) in a Perkin Elmer Analyst 2000 (Perkin Elmer, Waltham, MA, USA), using a flame as nebulizer with air-acetylene and a hollow cathode lamp. To determine sodium and potassium, CsCl was added as a matrix. For each element the calibration curves and the limits of detection (LOD) and quantification (LOQ) were determined according to Mateos-Aparicio *et al.* (2010) and Aldars-García *et al.* (2013).

All results are given on a dry mass (d.m.) basis.

### Soil samples

#### Collection.

Soil samples (upper 5 cm) were collected into Whirlpak bags using sterile techniques, and immediately frozen and kept frozen until analysed in Madrid.

#### Soil analysis.

Soil samples were thawed to room temperature and the moisture content was calculated by weighing before and after drying in an oven at 105 °C.

Analytical parameters were determined according to van Reeuwijk (2002). All chemicals were analytic-grade reagents obtained from Merck (Germany) and Panreac (Spain). All glassware used was washed with an aqueous solution of 1 % nitric acid for 24 h and rinsed with deionized type I water.

Soil Na, Cl and K were extracted by shaking soil aliquots (30 g) in (1:5) deionized water and filtered before the analysis. Na and K were quantified by flame atomic emission spectroscopy using a Sherwood 410 flame photometer.

Chloride was measured by ionic chromatography using a Metrohm 761 Compact IC. The separation was performed on a Metrosep A supp1 analytical column with an eluent composed of 3 mM of sodium carbonate. The flow rate was 1 mL min^−1^ and the injection volume was 20 μL. Two replications of each analysis were performed and mean values were used for calculations.

Ca was extracted by shaking 10 g of soil in 50 mL of 0.5 M K_2_SO_4_ for 30 min, filtered and quantified by flame AAS (Analytik Jena NovAA 300) (van Reeuwijk 2002).

### Statistical analysis

All statistical analyses were carried out using the statistics package XLSTAT, Addinsoft, USA. All comparisons were made using a one-way analysis of variance (ANOVA) with plant age (of substrate) or geographic groupings as independent variables and the dependent variables being element content (Na, sodium; Cl, chloride; K, potassium). When significant results (*P* < 0.05) were obtained from the ANOVA, then *post hoc* testing was carried out using Tukey’s HSD (honest significant difference) test. When age was used as the independent variable, the tests were made only with sites 2 to 5 (ages 7, 10, 19 and 34 years) because site 1 (age 3 yr) contains data for only a single species (*G. magellanica*). All graphs were prepared using Sigmaplot, SYSTAT Software, San Jose, CA, USA.

## Results

### *Gunnera magellanica* along the chronosequences

**Figure 2.**
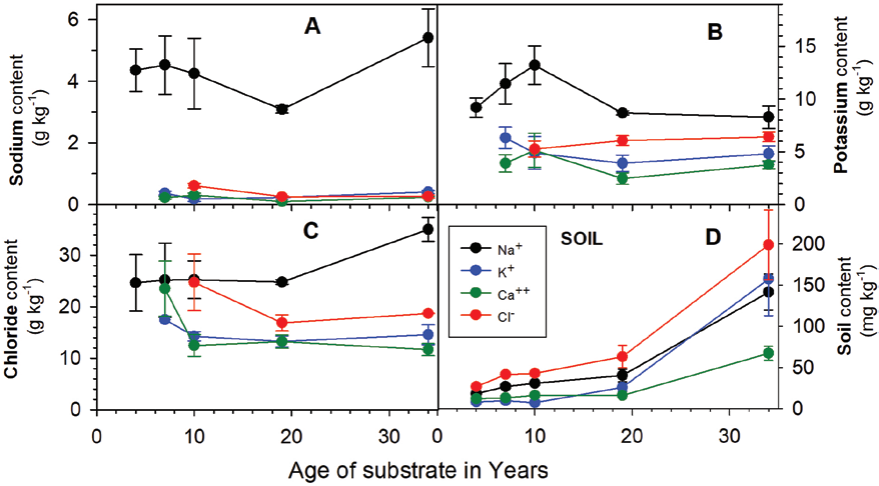
Sodium (A), potassium (B), chloride (C) contents in g kg^−1^ of leaf tissue (±1 SE) of *Gunnera magellanica* (black symbols and line), *Gaultheria mucronata* (blue symbols and line), *Nothofagus antarctica* (red symbols and line), *N. betuloides* (green symbols and line) along a chronosequence of increasing surface age in front of Pía Glacier, Tierra del Fuego, Chile. *X* axis is age of substrate in years after exposure by glacier retreat. Panel D shows the soil content of sodium, potassium, calcium and chloride (see inset) in mg kg^−1^ (±1 SE).

*Gunnera magellanica* along the chronosequence at Pía Glacier, Cordillera Darwin, Chile, had a mean leaf sodium content of 4.41 ± 0.40 g kg^−1^. This was about 15 times higher than that of the other plant species (*Gaultheria mucronata*, *N. betuloides* and *N. antarctica*) in which sodium levels were in the range 0.1 to 0.5 g kg^−1^ and well within the levels reported for normal glycophytes (Larcher 2003) (Table 1). This difference was highly significant (*F*_3, 78_ = 67.148, *P* < 0.0001) as there were no samples in which the sodium contents of *G. magellanica* leaves overlapped with that of the other species. Both analytical techniques (ICP/MS and ion chromatography) gave similar results. Soil sodium levels are low, ranging from 16.4 mg kg^−1^ at the youngest site to 141.5 mg kg^−1^ at the oldest site adjacent to the fjord (Fig. 2D) but, despite this increase in soil sodium content at the older site, there were no significant differences in sodium content for any of the species along the chronosequence (*F*_3, 72_ = 0.745, *P* = 0.529) (Fig. 2A). Similar elevated levels of sodium were also found in *G. magellanica* plants from a second chronosequence in front of Parry Glacier on the north side of the Cordillera Darwin and were once again about 15 times those of the other species that were present.

*Gunnera magellanica* along both chronosequences also had significantly (*F*_3, 166_ = 30.522, *P* < 0.0001) higher potassium than the other species present although the difference was not so marked as for sodium (8.47 versus 4.73 g kg^−1^; Table 1). Despite the higher levels of potassium, the Na:K molar ratios of symbiotic plants (Fig. 2; Table 1) were around 0.74 to 1.0, a magnitude higher than the 0.10 to 0.12 for non-symbiotic species but still much lower than values up to around 20 found in salt-challenged glycophytes (Glenn *et al.* 1999). Chloride was also significantly different between species (*F*_3, 39_ = 9.982, *P* < 0.0001) and was higher in *G. magellanica* than in *Gaultheria mucronata* and *N. betuloides*, but not compared to *N. antarctica* (*P* = 0.006 and 0.008; 26.99 versus 14.50, 15.23 and 20.29 g kg^−1^, respectively) (Fig. 2C; Table 1).

### *Gunnera magellanica* from other locations and in culture

Leaf samples from *G. magellanica* plants collected in the vicinity of Puerto Williams, Navarino Island, Chile, all had even higher sodium contents (Table 1). Puerto Williams lies on the south shore of the marine seaway Beagle Channel and plants nearer the shore have higher sodium contents (8.95 ± 1.89 g kg^−1^) than those more distant in the forest and the open tundra above the tree line (mean values of 5.67 to 6.98 g kg^−1^). Na:K ratios also fall from 3.58 near the coast to 1.69 at the tundra (Table 1).

*Gunnera magellanica* plants collected from near Puerto Williams, transplanted to greenhouses in Madrid and grown for 2 years with local water supply (see Methods) retained high sodium levels although significantly lower (*F*_1, 20_ = 30.097, *P* < 0.0001) than freshly collected plants (4.62 versus 8.23 g kg^−1^) and similar to the range found in this plant at the Pía and Parry glacier forelands. Cultivated plants had significantly higher chloride contents (40.34 versus 20.93 g kg^−1^, *F*_1, 23_ = 53.919, *P* < 0.0001) whilst potassium levels remained unchanged (*F*_1, 23_ = 0.0001, *P* = 0.977). *Gunnera magellanica* plants sourced in from plant nurseries in Scotland and Cornwall also all had elevated sodium (4.49 ± 0.16 g kg^−1^).

### Other *Gunnera* species and sites

Additional *Gunnera* species from natural habitats as well as from cultivated sites were also analysed: *G. manicata* from Lamorna, Cornwall, England, and *G. manicata*, *G. perpensa*, *G. hamiltonii* and *G. tinctoria* from Kew Gardens, London. All samples had elevated sodium contents that equalled or were higher than those from both glacial forelands in Chile (Table 1). The highest sodium content (7.61 ± 0.39 g kg^−1^) and Na:K ratio (2.79) were from *G. manicata* growing by a stream close to the sea shore at Lamorna, Cornwall. These species also had elevated potassium (mean 12.53 ± 3.02 g kg^−1^) with particularly high contents in *G. manicata* and *G. hamiltonii* from Kew Gardens (22.05 and 28.29 g kg^−1^, respectively). Mean chloride content was also very high at 33.03 ± 0.35 g kg^−1^ and reached over 51 g kg^−1^ in leaves of *G. manicata* at Lamorna and also from *G. hamiltonii* at Kew Gardens meaning that these plants were more than 6 % salt (potassium and sodium chloride; dry mass basis).

### Plants with the same functional trait—cyanobacterial symbiosis

We analysed samples from *A. rubra* from three sites in New Zealand and *A. filiculoides* (Fig. 1C) from a site in Spain. Sodium levels were significantly higher in the *Azolla* samples, regardless of source, in the range 10 to 16 g kg^−1^ and significantly more than in *G. magellanica* (*F*_1, 36_ = 42,809, *P* < 0.0001; Table 1). *Azolla* species also had significantly very high levels of potassium (*F*_1, 36_ = 38.181, *P* < 0.0001), 20.10 ± 1.87 g kg^−1^, and chloride (*F*_1, 23_ = 8.174, *P* < 0.009), 37.30 ± 2.53 g kg^−1^ (Table 1). Root tissue containing cyanobacterial symbionts of *C. revoluta* (example *Cycas* species in Fig. 1D) had a sodium content of 2.52 ± 0.34 g kg^−1^ and, although this is lower than leaf levels in *Gunnera* and *Azolla*, it is still higher than typical glycophytes and 10 times more than the non-symbiotic plants analysed here (Table 1).

## Discussion

Our results support a novel grouping of plants that maintain sodium, chloride and potassium contents that are much higher than those in other species growing at the same locations and without elevated salt levels in the external medium. Outwardly these plants appear normal and show no signs that they accumulate salt.

*Gunnera magellanica* growing along chronosequences in Tierra del Fuego had leaf sodium contents that were around 15 times higher than for other species growing at the same site and the levels were maintained for the same species at other locations. When analyses were extended to other *Gunnera* species, they were all found to have similarly high sodium contents. *Gunnera* is an unusual genus in that all members have the cyanobacterium *Nostoc* as an endocellular symbiont in special glands at the base of the petioles (Rai *et al.* 2000; Osborne and Berman 2009; Fernández-Martínez *et al.* 2013). Possession of cyanobacterial symbionts is a rare functional trait in plants (Rai *et al.* 2000) with one of the better known examples being the fern genus *Azolla* in which the symbiotic cyanobacteria are in cavities within the leaves. The *Azolla* species analysed had even higher sodium levels than the *Gunnera* species and these are supported by occasional, but unremarked, reports in the literature of high sodium levels in *Azolla*, 15.25 g kg^−1^ (Buckingham *et al.*, 1978) and 9.2 g kg^−1^ (Lumpkin and Plucknett 1980). Species in the gymnosperm genus *Cycas* have cyanobacteria in specialized coralloid roots (Lindblad 2009) and we found this tissue to also have elevated sodium levels and, although lower than *Gunnera* and *Azolla* they are still higher than for typical glycophytes and 10 times more than the non-symbiotic plants analysed here (Table 1). We suggest that other symbioses that we have not analysed, such as cyanophyte-bryophyte symbioses (Adams and Duggan 2008), will also show high sodium contents.

Several lines of evidence suggest that the elevated sodium levels might be obligate in this symbiotic association: (i) they occurred in the absence of external elevated soil salt levels and were independent of levels in the soil; (ii) other plants growing together with *G. magellanica* had normal sodium levels as expected for typical glycophytes; (iii) all *Gunnera* species showed the same high sodium levels regardless of where they were growing, whether in natural or cultivated habitats. *Azolla* species showed the same behaviour; (iv) *G. magellanica* plants cultivated for 2 years in a greenhouse on normal tap water retained their high sodium levels. Further investigations, especially clarifying the possible functional significance, are needed to clarify this.

The coincidence of obligate high sodium content with the presence of cyanobacterial symbiosis, the latter being extremely rare in the plant kingdom, strongly suggests that these two traits are in some way linked. The basis of the linkage is not clear although there are some possibilities. The simplest is that the presence of the salt could reduce growth of fresh water green algae thus preventing their proliferation in or near the cyanobacterial sites where there might be expected to be elevated nitrogen availability. Certainly cyanobacteria are able to withstand saline conditions (Reddy *et al.* 1989) whilst most fresh water algae are sensitive to salt. There is also a possible link between sodium levels and degree of exposure of the cyanobacteria to environmental light. Higher light would encourage algal growth and light levels are probably highest in *Azolla* and lowest in *Cycas* roots. A second possibility is that the salt initiates the production by the cyanobacteria of metabolites to obtain an osmotic balance. It has been shown that the export of sucrose, the major compatible metabolite in cyanobacteria with low salt tolerance, can be up-regulated by increasing external salt concentrations in a genetically modified strain of *Synechococcus elongatus* (Ducat *et al.* 2012; Du *et al.* 2013). A similar situation might occur with the export of a nitrogen compound being stimulated by the external salt concentration. Other less likely explanations related to improved functioning of the nitrogen fixation system are: first, sodium, at low levels, is an essential element for some cyanobacteria and certainly causes increases in growth (Allen and Arnon 1955; Thomas and Apte 1984). Second, chloride may be important as high levels can suppress nitrification by external associated bacteria and this would prevent loss of excreted ammonia by conversion to nitrate (Xu *et al.* 2000). The above suggestions are not exclusive and the possibility remains of some as yet unknown physiological drivers.

The elevated sodium levels occur in conjunction with higher potassium and chloride contents although the increase is not so large being around double rather than 15 times. Despite the greatly increased sodium content, the presence of more potassium means that the Na:K ratio, although reaching 3.58 compared to 0.1 in non-symbiotic plants, is still much less than the ratios in salt-challenged glycophytes (Glenn *et al.* 1999). *Gunnera manicata* and *G. hamiltonii* from Kew Gardens had particularly high potassium (22.05 and 28.29 g kg^−1^, respectively) and chloride contents (over 51 g kg^−1^) as did also *Azolla* species (potassium, 20.10 ± 1.87 g kg^−1^, and chloride, 37.30 ± 2.53 g kg^−1^) (Table 1). These plants were more than 6 % salt (potassium and sodium chloride; dry mass basis) and chloride levels are close to those rated as toxic for most plants (Xu *et al.* 2000).

## Conclusions

We have found a novel group of plants that appear to obligately accumulate salt in the absence of an environmental challenge. The group is taxonomically diverse including angiosperms (*Gunnera* spp.), gymnosperm (*Cycas*) and a fern (*Azolla* spp.) but all of them share a rare functional trait, they form a symbiosis with a cyanobacterium that carries out nitrogen fixation. The reason why salt accumulation is linked to the symbiosis still needs to be explained; however, we hypothesize that either metabolic forcing to drive nitrogen transfer to the plant or sugar uptake by the cyanobacteria are possibilities. Despite the high internal sodium levels, these species look and behave like normal glycophytes and the literature suggests that they cannot tolerate elevated environmental salt. This is best known for *Azolla* species (Rai *et al.* 2006; Carrapiço 2010). It appears, therefore, that there may be no apparent linkage between the high internal sodium, potassium and chloride and ability to handle external salt. The findings suggest future lines of research to better understand this rare, but highly effective, symbiosis.

## Sources of Funding

This research was funded by Spanish Economy Ministry (CTM2015-64728-C2-1-R).

## Contributions by the Authors

S.C. prepared samples and performed ICPS/MS analyses; L.G.S., T.G.A.G. and A.P. designed the study, analysed the data, carried out field work and wrote the paper; D.S. and S.M. performed the leaf analyses in Madrid; M.A.C. and M.T.C.C. carried out the soil analyses, M.A.-C. carried out additional field work and plant collection; R.R. organized field logistics, selected field sites and arranged required permissions and permits. All authors discussed the results and commented on the manuscript.

## Conflicts of Interest

None declared.
